# BDNF/TrkB Signaling in the Brain–Kidney Axis Under Functional Stress

**DOI:** 10.3390/biology15090696

**Published:** 2026-04-29

**Authors:** Anna Beknazarova, Victoria Kuvaeva, Maxim Baltin, Kerim Mutig, Alexander Bobylev

**Affiliations:** 1 Scientific Center of Genetics and Life Sciences, Sirius University of Science and Technology, 354340 Sirius, Russia; beknazarova.am@talantiuspeh.ru (A.B.); kuvaeva.vy@talantiuspeh.ru (V.K.); baltin.me@talantiuspeh.ru (M.B.); 2 Institute of Theoretical and Experimental Biophysics, Russian Academy of Sciences, 142290 Pushchino, Russia

**Keywords:** brain-kidney axis, brain-derived neurotrophic factor (BDNF), TrkB (*NTRK2*), actin cytoskeleton remodeling, podocytes, extreme physical exercise

## Abstract

Brain-derived neurotrophic factor (BDNF) and its high-affinity receptor tropomyosin receptor kinase B (TrkB) are classically associated with neuroplasticity, but increasing evidence suggests a broader role for BDNF/TrkB signaling in systemic stress adaptation beyond the central nervous system. Strenuous exercise is a model of functional stress that may become a clinically relevant renal challenge under conditions such as dehydration, heat stress, vascular vulnerability, and repeated exposure. Neuroendocrine stress activation, hemodynamic perturbations, and cytoskeletal instability are key factors that may contribute to glomerular barrier dysfunction in this setting. BDNF biogenesis is complex, and circulating BDNF largely reflects platelet-associated pools and context-dependent release. At the tissue level, BDNF/TrkB signaling can activate actin-regulatory pathways involved in cellular resilience. The podocyte is of particular interest because its actin-dependent architecture functionally parallels that of neurons and is essential for maintenance of the glomerular filtration barrier. Within this framework, BDNF/TrkB signaling may stabilize podocyte actin dynamics, reduce foot process effacement, and attenuate proteinuria. The present review focuses on the brain–kidney axis and the potential renoprotective role of BDNF/TrkB signaling, while highlighting major knowledge gaps regarding BDNF availability to glomerular cells, isoform-specific TrkB actions, and causal inference in humans exposed to repeated exercise-related renal stress. However, current human evidence is insufficient to define the dominant source and delivery route of BDNF to glomerular cells during exercise-related renal stress. Therefore, BDNF/TrkB is discussed here as a candidate modulatory/resilience pathway rather than an established causal driver.

## Simple Summary

Although exercise is usually beneficial, extreme exertion under dehydration, heat stress, or vascular vulnerability may challenge kidney function. In this review, we explore whether brain-derived neurotrophic factor (BDNF), a molecule best known for its role in the nervous system, may also contribute to kidney protection during such stress. We focus on podocytes, specialized cells that are essential for maintaining the kidney’s filtration barrier. Current evidence suggests that BDNF signaling may help preserve podocyte structure and function. However, key questions remain unresolved, including how much BDNF reaches the kidneys in humans, which receptor forms are most relevant, and whether modulation of BDNF can reduce exercise-induced kidney injury. A better understanding of these mechanisms may improve risk assessment and support the development of strategies to protect kidney function in physically stressed individuals.

## 1. Introduction

The nervous system and the kidneys are tightly integrated through central control of renal function, which is essential for maintaining fluid homeostasis, electrolyte balance, and systemic hemodynamics. Beyond the classical brain–kidney axis mediated by renal innervation and the hypothalamic–pituitary axis (HPA), humoral mediators such as neurotrophic factors, including brain-derived neurotrophic factor (BDNF), are increasingly considered potential modulators of renal regulation despite their traditional association with the nervous system [1,2,3,4,5]. Brain–kidney communication is bidirectional: the central nervous system (CNS) regulates renal blood flow, glomerular filtration, and tubular transport [6,7], whereas the kidney signals its functional status to the brain via sensory (afferent) pathways, enabling adaptive neurohumoral responses [8,9].

Functional stress (e.g., extreme physical exercise), robustly activates this axis and induces marked autonomic shifts, blood-flow redistribution, and metabolic and hemodynamic remodeling in renal tissue [10,11,12]. Here, we use “functional stress” as an umbrella term for acute high-demand physiological states that impose combined hemodynamic and metabolic load on the kidney. Extreme endurance exercise is used as the primary exemplar, whereas heat stress and hypohydration/dehydration are treated as co-stressors that amplify the exposure. However, the central mechanisms supporting adaptation to this type of stress remain incompletely defined [13,14]. In parallel, neurotrophic factors, particularly BDNF, are well-established mediators of stress-related neuroplasticity. Specifically, BDNF supports neuronal survival and promotes dendritic spine growth, enhances synaptic transmission, and facilitates long-term potentiation [15,16]. Importantly, BDNF/TrkB signaling has also been reported in peripheral tissues, including the kidney, where BDNF and its receptors are expressed (notably in collecting duct structures) and may contribute to renal functional regulation [17]. The BDNF/TrkB cascade has further been implicated in pathological remodeling, including renal fibrosis, supporting a broader view of BDNF as a systemic mediator of inter-organ communication [18]. Within the HPA, BDNF modulates corticotropin-releasing hormone (CRH) regulation and influences stress reactivity [19].

Importantly, BDNF is positioned at the interface between central stress circuits and peripheral organ physiology. Within the hypothalamic–pituitary axis (HPA), BDNF has been linked to the regulation of corticotropin-releasing hormone (CRH) and stress reactivity, providing a mechanistic entry point for neuroendocrine modulation of systemic hemodynamics during functional stress [19]. In peripheral tissues, BDNF/TrkB signaling is not restricted to the nervous system. In the cardiovascular context, BDNF is considered a trophic factor that supports cardiomyocyte viability and may confer protection under ischemic conditions [20,21]. In the kidney, BDNF/TrkB signaling has been reported not only in functional regulation but also in pathological remodeling, including renal fibrosis, underscoring the context-dependent nature of this pathway in target organs [18]. Brain–kidney communication is bidirectional. Central autonomic and neuroendocrine outputs shape renal perfusion and filtration, whereas renal perturbations can engage afferent pathways and humoral cues that influence central stress responses. Together, these observations shape a brain–kidney axis framework in which BDNF-related signaling can be discussed both as a central modulator of stress physiology and as a local tissue pathway with potentially protective or maladaptive consequences depending on the injury milieu [18,19].

In the circulation, BDNF is predominantly platelet-associated and can be released in response to stress and physical exercise [22,23]. Local expression of BDNF and TrkB has been reported in renal compartments relevant to barrier integrity: BDNF has been detected in podocytes and tubular epithelial cells, and TrkB expression has been described in vascular endothelium, suggesting potential roles in cytoskeletal remodeling and filtration barrier maintenance [17,23,24]. In the cardiovascular system, BDNF is considered a trophic factor that supports cardiomyocyte viability and can confer protection under ischemic conditions [20,21]. Despite this expanding evidence base, the role of BDNF in brain–kidney communication during extreme physical exertion remains poorly characterized. Moreover, kidney-specific intracellular mechanisms, particularly in podocytes, are rarely addressed.

This review summarizes nervous system–kidney interactions, considers BDNF as a candidate mediator of this crosstalk, and examines how BDNF-dependent signaling may contribute to renoprotection. We focus on mechanisms preserving podocyte structural integrity under functional and chronic stress. We first outline the classical brain–kidney axis and its hormonal regulation, then summarize BDNF biology and TrkB receptor distribution. Next, we review evidence for renal BDNF/TrkB signaling under stress. Finally, we compare two actin-dependent specialized cell types, the neuron and the podocyte, to discuss how cytoskeletal dynamics may shape stress resilience. Throughout this review, we distinguish between mechanistic evidence from experimental models and largely associative observations in humans. To interpret BDNF/TrkB signaling as a candidate modulator of podocyte resilience during functional stress, it is necessary to distinguish between BDNF sources, circulating forms, delivery routes, and target cells. First, BDNF could act through local renal production (tubular epithelium, vascular endothelium, and/or glomerular cells) with paracrine signaling to the filtration barrier [17,23,24]. Second, circulating BDNF is largely influenced by platelet-associated pools and context-dependent release, and therefore blood measurements do not directly report the free fraction available for tissue signaling [22,25,26,27]. Third, an indirect central route is plausible in which BDNF shapes neuroendocrine stress reactivity and sympathetic outflow, thereby modifying renal hemodynamics and the mechanical load on the glomerular filtration barrier [19]. Regarding access to glomerular cells, a key unresolved issue is whether podocytes primarily respond to locally produced BDNF or to the bioavailable (platelet-independent) free fraction via endothelial transport mechanisms (e.g., receptor-mediated transcytosis) and/or other routes. Passive filtration of the free fraction is also a theoretical possibility but remains constrained by size/charge selectivity of the barrier. This uncertainty is highlighted as a major limitation of the current evidence base that frames priorities for causal testing [23,24].

## 2. When Functional Stress Becomes a Renal Threat

It is useful to delineate when strenuous exercise shifts from a physiological challenge to a clinically meaningful renal stressor. The risk of an exercise-associated acute kidney injury (AKI) phenotype increases with extreme workload combined with heat stress and dehydration, insufficient recovery between competitions or heavy training blocks, NSAID use during an event, intercurrent illness, and episodes of rhabdomyolysis [28,29,30]. Clinically, the key issue is often not a single post-race episode but recurrence. First, even a single episode of AKI is associated with a higher long-term risk of chronic kidney disease (CKD) and adverse long-term outcomes in longitudinal cohorts [31]. Second, repeated AKI episodes are linked to additional risk, consistent with a cumulative-injury framework in which recurrent insults accelerate kidney function decline and worsen prognosis [32,33].

Vascular status further modifies vulnerability. Increased arterial stiffness and elevated pulse pressure are linked to albuminuria and may amplify the pulsatile load transmitted to the renal microcirculation and glomerulus, thereby increasing podocyte susceptibility to hemodynamic stress during extreme exercise [34,35,36]. Increased pulsatile loading is likely to elevate mechanical strain at the glomerular filtration barrier, challenging the actin cytoskeleton that maintains podocyte foot process architecture and predisposing to effacement. This coupling between hemodynamic stress and cytoskeletal remodeling provides a rationale to consider candidate resilience pathways converging on actin regulation, including BDNF/TrkB signaling. Against this background, the central question is which molecular pathways support cytoskeletal stability and the resilience of the glomerular filtration barrier under repeated functional stress. In this context, BDNF/TrkB signaling is a plausible candidate mechanism in renal cells, including podocytes, given its established role in actin-dependent structural plasticity in the nervous system. This hypothesis is supported by mechanistic podocyte injury models showing TrkB-dependent actin stabilization and reduced proteinuria [23], although translation to human exercise settings remains to be established. However, the contribution of TrkB-dependent F-actin-stabilizing programs under the combined burden of arterial stiffness, recurrent AKI, and systemic inflammation remains hypothetical and requires direct experimental validation.

## 3. BDNF Biosynthesis, Cellular Distribution, and Signaling

BDNF biosynthesis begins with transcription of the BDNF gene, which uses multiple promoters and alternative 5′ transcript variants. Translation yields preproBDNF, which is converted to proBDNF after signal peptide cleavage. ProBDNF is then proteolytically processed into mature BDNF (mBDNF) either intracellularly along the secretory pathway or extracellularly after secretion [37,38]. The prodomain and the functional state of the cell influence sorting, packaging, and release. This distinction is mechanistically important because proBDNF and mBDNF can bias signaling toward different receptor branches, namely p75NTR-dependent and TrkB-dependent pathways [39]. Mature BDNF preferentially activates TrkB and is typically linked to pro-survival and plasticity programs. In contrast, proBDNF more often signals through p75NTR, frequently in complex with the co-receptor sortilin, and can promote synaptic weakening, reduced neurite outgrowth, process retraction, and, in some contexts, pro-apoptotic programs. Accordingly, the balance between proBDNF and mBDNF is a key determinant of the direction of the cellular response [39,40].

**Figure 1:**
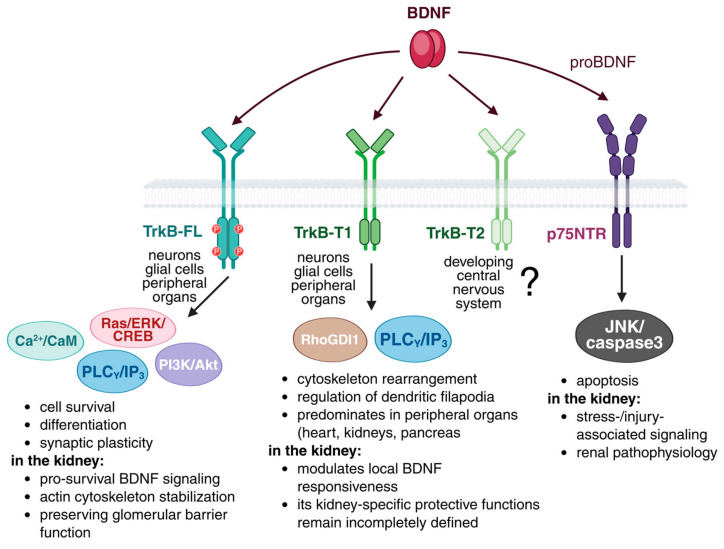
BDNF receptor signaling pathways. Mature BDNF binds full-length TrkB (TrkB-FL), mainly expressed in neurons and glial cells, and activates the Ras/ERK/CREB, PI3K/Akt, and PLCγ/IP3 pathways, with additional Ca^2+^/calmodulin-dependent signaling. These pathways regulate cell survival, differentiation, synaptic plasticity, and Ca^2+^ homeostasis. Truncated TrkB isoforms (TrkB-T1 and TrkB-T2) are expressed in a tissue-specific manner, including in peripheral organs such as the heart and kidneys, and have been reported in some contexts to be associated with cytoskeletal remodeling, filopodia formation, and dendritic organization. Putative mediators include RhoGDI1 and PLCγ/IP3, although isoform-specific coupling remains incompletely defined. In contrast, proBDNF preferentially binds p75NTR and activates JNK- and caspase-3-dependent signaling linked to apoptosis. In the context of renal cells, this schematic should be interpreted as a conceptual/hypothetical framework rather than an established cell-type-specific pathway map. The “?” indicates the limited evidence available for TrkB-T2-specific functions.

Binding of the mature BDNF dimer to TrkB promotes receptor dimerization and autophosphorylation of tyrosine residues within the intracellular domain (Figure 1). These phosphotyrosines recruit adaptor proteins such as Shc, FRS2, and PLC-γ and initiate three major cascades [41,42]. In the MAPK and ERK pathway, the Shc, Grb2, and SOS complex activates Ras and engages Raf, MEK, and ERK1/2. Phosphorylated ERK translocates to the nucleus and activates CREB-dependent transcriptional programs that support axonal and dendritic growth, differentiation, and long-term maintenance of synaptic contacts [43]. In the PI3K and Akt pathway, PI3K activation promotes Akt phosphorylation. This signaling suppresses pro-apoptotic proteins such as Bad and engages mTOR-dependent pro-survival programs, supporting protein synthesis and structural remodeling [44]. In the PLC-γ pathway, recruitment of PLC-γ drives PIP2 hydrolysis and generates DAG and IP3. IP3 induces Ca^2+^ release from intracellular stores, and calcium transients activate CaMKII and CaMKIV, which support CREB-dependent transcriptional programs sustaining synaptic plasticity [45,46].

Two features largely shape the in vivo consequences of the BDNF and TrkB system. First, the *NTRK2* gene encoding TrkB produces multiple receptor isoforms. These include full-length TrkB-FL containing the intracellular tyrosine kinase domain and truncated variants such as TrkB-T1 and TrkB-T2 that lack tyrosine kinase activity [47,48,49]. TrkB-FL is classically associated with canonical survival and plasticity programs. Truncated isoforms are broadly expressed across cell types and can limit TrkB-FL signaling through heterodimerization, regulate ligand availability by sequestration, and in some contexts have been reported to engage tyrosine kinase-independent responses, including calcium-related signaling events, although this appears context- and cell type-dependent [50]. TrkB-T1 is widely expressed outside the CNS and is frequently reported as a prominent truncated TrkB variant in several peripheral organs, including the kidney [48]. In renal tissue, the most informative evidence comes from studies that directly examined multiple isoforms, noting that isolated glomeruli represent a mixed-cell preparation. Analyses of isolated glomeruli have detected both TrkB-FL and a truncated TrkB isoform [23], supporting the presence of multiple receptor forms within glomerular tissue and motivating isoform-specific functional testing.

Second, tissue distribution determines which cells can respond to BDNF. In the CNS, TrkB is broadly expressed in circuits implicated in learning, memory, and behavior, with prominent representation in the hippocampus and cerebral cortex and detectable expression across additional regions [41,51,52]. TrkB is also detected in astrocytes, predominantly as truncated isoforms, whereas evidence for intrinsic TrkB expression in microglia remains mixed. Nevertheless, BDNF has been reported to modulate microglial inflammatory phenotypes in selected contexts [53,54,55]. In the peripheral nervous system, TrkB is expressed in sensory and motor neurons, including dorsal root ganglia, cranial ganglia, and spinal motor neurons, where BDNF/TrkB signaling supports neuronal survival and adaptive responses after injury [56,57].

In non-neuronal tissues, TrkB expression is generally lower than in the CNS and is strongly cell type dependent. TrkB has been reported in glomus cells of the carotid body and implicated in ventilatory responses to hypoxia [58]. TrkB has also been described in reproductive tissues, including ovarian compartments, where signaling is linked to folliculogenesis and oocyte viability [59,60]. In vascular biology, BDNF and TrkB signaling has been described in endothelial cells and linked to nitric oxide dependent effects and endothelial reactivity [61]. TrkB expression has also been reported in renal tissue, including glomerular-related contexts. However, cell type– and isoform-specific functions in podocytes and neighboring glomerular cells remain incompletely defined [23,24].

In the kidney, *NTRK2* is detectable, and experimental data support protective effects on podocyte structural integrity. However, in vivo operating conditions remain unclear. It is uncertain which combinations of stressors, inflammatory and hemodynamic regimes, metabolic states, or pharmacological exposures modulate local BDNF and TrkB responses in the glomerulus. Cell type specificity within the glomerular filtration apparatus is also insufficiently resolved, as is the stability of these responses across injury phenotypes. Despite these uncertainties, existing mechanistic studies suggest that BDNF/TrkB signaling may help podocytes withstand structural stress. In this model, BDNF promotes TrkB-dependent trophic effects on podocyte processes and engages actin-remodeling nodes through the LIMK1 and cofilin axis. This includes regulation of microRNA-134 and microRNA-132 and promotes actin polymerization. These pathways may facilitate repair in experimental models of proteinuria and focal segmental glomerulosclerosis [23].

Taken together, these findings suggest a possible link between systemic stress responses and the mechanical burden experienced by the glomerular filtration barrier. Sympathoadrenal activation, inflammatory signals, hypoxia, and endothelial dysfunction can reshape vascular reactivity and the intraglomerular hemodynamic milieu, thereby increasing mechanical and metabolic load on the glomerular filtration barrier. Against this background, a local BDNF and TrkB system is a plausible resilience module that promotes stabilization of the actin scaffold within podocyte processes. Within the brain–kidney axis framework, systemic mediators primarily shift the hemodynamic regime, while local BDNF and TrkB signaling may enhance podocyte tolerance to the resulting increase in load.

## 4. Central Effects of BDNF

BDNF is a central regulator of neuronal survival and activity-dependent plasticity in the CNS. TrkB distribution across CNS regions and glial compartments is summarized in Section 3 [41,51,52,53,54,55]. At the synaptic level, BDNF supports dendritic spine remodeling and long-term potentiation, thereby contributing to adaptive circuit reorganization [15,16,52].

Beyond its classical neuroplasticity role, BDNF is implicated in central control of systemic stress responses, including blood pressure regulation and sympathetic outflow [19]. Consistently, BDNF deficiency has been linked to altered HPA reactivity, although both hyperreactive and hyporeactive phenotypes have been reported. These apparently opposite outcomes likely reflect differences in experimental context, including developmental versus adult manipulations, region-specific circuitry, stressor characteristics (acute or chronic), and compensatory adaptations within glucocorticoid feedback and sympathetic pathways [62]. Accordingly, across models BDNF is best viewed as a modulator of stress-circuit set points rather than a strictly unidirectional driver of HPA output. Mechanistically, BDNF has been reported to modulate corticotropin-releasing hormone (CRH) expression in the hypothalamic paraventricular nucleus and to interact with glucocorticoid-related regulatory loops, with the CREB co-activator CRTC2 proposed as one potential switch shaping these responses [19,57,63,64].

In the brain–kidney axis context, an unresolved question is whether peripheral BDNF can influence central circuits. Experimental work suggests that BDNF can cross the blood–brain barrier via a saturable transport mechanism [65], although its physiological relevance in humans remains debated. BBB permeability has been reported to increase under severe or prolonged systemic stress in some experimental contexts, which could facilitate access of peripheral factors to the CNS [65]. If such permeability shifts occur, they may also promote neuroinflammatory signaling by permitting greater entry of plasma proteins and inflammatory mediators. However, the magnitude and physiological relevance of this route in humans remain uncertain [66,67,68,69].

## 5. Peripheral Effects of BDNF

Beyond the CNS, a substantial fraction of circulating BDNF is platelet-associated and can be released upon platelet activation during vascular or inflammatory stress and intense physical exertion [22,24]. The origin of platelet-associated BDNF remains not fully resolved and may reflect non-mutually exclusive mechanisms, including endogenous loading during megakaryocyte/platelet biogenesis and acquisition from the extracellular milieu via uptake/binding and platelet–endothelium interfaces [70,71]. Accordingly, serum BDNF concentrations are typically higher than plasma values because coagulation triggers platelet degranulation, and interpretation is strongly dependent on the sampled matrix [25,26,27]. Therefore, blood BDNF measurements do not directly quantify the free, bioavailable fraction relevant for tissue signaling.

In selected peripheral contexts, BDNF/TrkB signaling has been reported to support stress-resilience programs in a context- and cell type-dependent manner, including cardiomyocyte viability under ischemic conditions [20,21] and endothelial barrier/junctional stability under inflammatory or hypoxic challenges [61]. These considerations motivate a cautious framework in which circulating BDNF is interpreted primarily as a context-dependent readout of platelet activation and systemic stress, while local BDNF/TrkB signaling may contribute to tissue-specific adaptive responses.

## 6. Renal Effects of BDNF

Available evidence indicates that BDNF mRNA and protein are present in multiple nephron segments and renal vascular structures. BDNF and its receptors are expressed in the kidney, particularly in collecting duct structures, where they may contribute to regulation of renal function and the urine-concentrating mechanism [17,72,73]. BDNF has also been detected in podocytes and tubular epithelial cells, whereas TrkB expression has been reported in renal vascular endothelium, suggesting potential roles in filtration barrier integrity and cytoskeletal remodeling [17,23,24]. Across these renal compartments, available studies have reported predominantly protective effects in stress contexts, including preservation of endothelial barrier integrity, reduced vulnerability to hypoxic and oxidative stress, stabilization of intercellular junctions, and involvement in local repair processes [3,17,74,75,76,77].

In podocytes, BDNF immunoreactivity has been reported in the cell body and major processes. Podocyte-focused studies most consistently report predominant expression of truncated TrkB isoforms (particularly TrkB-T1), while detection of TrkB-FL appears more variable across experimental contexts. Importantly, isoform detection depends on the biological level examined. Isolated glomeruli represent a mixed-cell preparation (podocytes, endothelial, and mesangial cells) in which both TrkB-FL and truncated variants can be detected [23], whereas podocyte-focused datasets more consistently report predominant truncated TrkB (particularly TrkB-T1) with variable TrkB-FL detection depending on species, injury context, and experimental system [17,50,75,78,79]. In podocyte models, BDNF/TrkB signaling has been associated with calcium-dependent responses and actin cytoskeletal remodeling, although isoform-specific functional attribution (e.g., TrkB-T1 or TrkB-FL) remains limited [50,75,78,79]. Mechanistic studies link BDNF/TrkB signaling to an actin-stabilizing program centered on LIMK1–cofilin regulation. In this framework, miR-134 acts as a negative regulator of LIMK1 translation. Therefore, BDNF-associated downregulation of miR-134 can relieve repression of LIMK1, increasing LIMK1 availability. In parallel, miR-132 upregulation has been reported in the same setting and may act in a coordinated manner to support cytoskeletal remodeling programs. Activated LIMK1 phosphorylates cofilin, suppresses actin depolymerization, and shifts the balance toward F-actin accumulation. In vitro, BDNF increases the number and length of podocyte processes, with the strongest effects in primary cultures during the first 24 h of incubation [23]. In models of glomerular injury, TrkB-dependent BDNF signaling has been linked to restoration of the podocyte actin cytoskeleton and reduced proteinuria [23]. Because cytoskeletal remodeling is context-dependent, sustained or non-physiological enhancement of BDNF/TrkB signaling could, in principle, promote maladaptive actin reorganization or narrow the adaptive range of podocytes under fluctuating mechanical demands. This is revisited in Translational Perspectives [23,80]. Conversely, experimental inhibition or genetic downregulation has been associated with podocyte dedifferentiation, apoptosis, and morphogenetic defects, supporting the notion that physiological BDNF levels contribute to nephron integrity and development [17].

A key unresolved issue is how podocytes gain access to BDNF, through local synthesis, delivery from the circulation, or both. Mature BDNF is ~13–14 kDa as a monomer and ~27 kDa as a dimer, suggesting that molecular size alone is unlikely to be the sole limiting factor for the free fraction under some conditions. However, glomerular handling depends not only on size but also on charge and interactions with negatively charged components of the filtration barrier. Given that BDNF is a basic protein, electrostatic constraints may limit bioavailability even when molecular size is permissive. Accordingly, the dominant route by which BDNF reaches glomerular cells (local renal production versus vascular/endothelial transport mechanisms and other pathways) remains insufficiently defined and should be treated as a major limitation of current models [23,24,61].

## 7. BDNF as a Biomarker of Kidney Damage

BDNF-related readouts have been proposed as candidate markers of renal stress and podocyte involvement, but the current evidence base remains limited and rests largely on a small number of mechanistic/clinical studies, with [23,24] representing a substantial fraction of the direct urinary-sediment transcript evidence. In CKD cohorts, BDNF and TrkB-related transcripts detected in urinary sediment have been reported to correlate with markers of nephron injury (e.g., KIM-1) and podocyte-related transcripts (e.g., NPHS1), suggesting that BDNF-axis activity may track nephron stress states [23,24].

Notably, urinary sediment BDNF mRNA has been reported to correlate negatively with uACR [24]. This association can be interpreted in at least two biologically plausible ways. One interpretation is that higher urinary BDNF-axis activity reflects better preservation of glomerular barrier integrity or a more effective local protective program. An alternative interpretation is that increased urinary BDNF mRNA reflects a compensatory stress response within nephron cells and/or shifts in the composition of urinary sediment during injury, which may not straightforwardly indicate barrier preservation.

More broadly, circulating BDNF is strongly shaped by platelet-associated pools and sampling methodology, complicating causal interpretation of blood BDNF as an effector signal for renal cells [22,25,26,27]. Accordingly, BDNF is best framed at present as a context-dependent candidate biomarker that may reflect systemic stress/platelet activation, local renal responses, or both, rather than as a validated causal mediator of kidney injury in humans [23,24]. Discriminating between these possibilities will require designs that jointly assess blood matrices (including platelet-poor plasma), urinary sediment transcripts, and podocyte-centered endpoints across defined stress exposures. Longitudinal sampling across recovery trajectories will be particularly informative for establishing directionality, and BDNF-related readouts should therefore be interpreted as candidate biomarkers pending prospective validation with podocyte-centered endpoints.

## 8. Neuron and Podocyte: A Shared Actin-Dependent Organizational Principle

**Figure 2:**
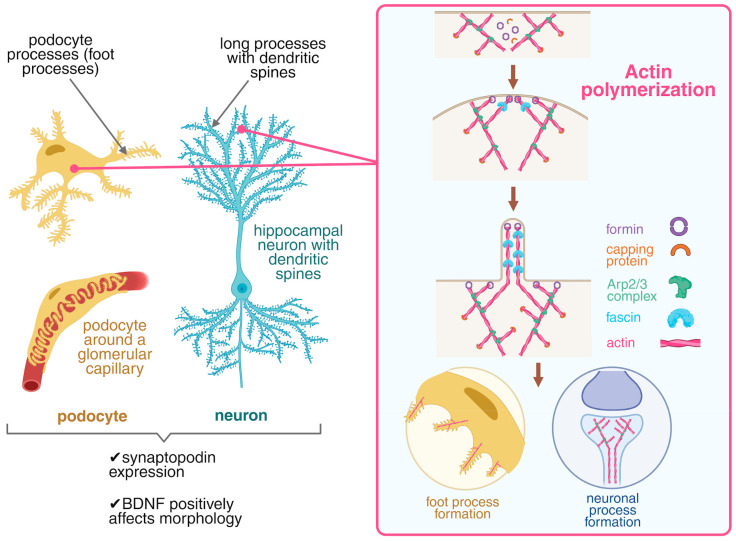
BDNF as a shared regulator of actin-dependent morphology in neurons and podocytes. The left panel shows conceptual parallels between neuronal dendritic spines and podocyte foot processes, two actin-rich protrusions that depend on dynamic actin remodeling and share regulators such as synaptopodin. The right panel outlines a proposed mechanism by which BDNF/TrkB signaling promotes actin polymerization via the Arp2/3 complex and formins, supporting the formation and stabilization of actin-based protrusions. In neurons, this contributes to neurite outgrowth. In podocytes, it may help preserve or restore foot processes. The neuron–podocyte analogy is presented as a conceptual framework supported by experimental model evidence, but it does not imply direct causal equivalence across tissues.

Podocytes share striking morphological and molecular similarities with neurons (Figure 2), supporting the idea that both cell types can be examined within a common framework of cytoskeletal organization and the mechanisms that maintain highly specialized cellular processes [81,82].

Both cell types are highly differentiated and are typically post-mitotic, or at least exhibit very limited proliferative capacity. Their larger processes (dendrites and major cellular projections), rely primarily on microtubule-based scaffolding, whereas finer specialized structures, such as neuronal dendritic spines and podocyte foot processes, depend critically on actin cytoskeletal dynamics [83,84]. Disruption of the organized actin network leads to structural simplification. In neurons, this manifests as spine loss and a simplified dendritic arbor, whereas in podocytes it results in foot process effacement accompanied by proteinuria [85,86]. In this context, BDNF/TrkB is a plausible shared regulator of the mechanical robustness of these cells because its downstream signaling converges on key nodes that control actin polymerization.

Below we summarize the main mechanisms through which BDNF may increase the resilience of these actin-dependent structures.

### 8.1. Small Rho GTPases

TrkB activation engages Rac1 and Cdc42, two central regulators of actin dynamics [87]. Importantly, these responses reflect crosstalk with RhoA-driven actomyosin contractility, which counterbalances Rac1/Cdc42 activity and is highly relevant to podocyte stability under mechanical stress. Cdc42 promotes the initiation of new protrusions (filopodia) and de novo actin assembly, whereas Rac1 drives peripheral actin polymerization and generates a dense cortical network that supports the volume and stability of dendritic spines and podocyte foot processes. In neurons, these dynamics are reflected in dendritic spine growth and stabilization. In podocytes, they support remodeling and reorganization of the actin network. Notably, the net outcome depends on the balance among small GTPase activities.

### 8.2. The LIMK1–Cofilin Axis

Cofilin is an actin-binding protein that disassembles F-actin and increases filament turnover, which can promote process collapse or effacement. Upon TrkB activation, BDNF engages LIMK1, which phosphorylates cofilin and suppresses its actin-depolymerizing activity [23]. As a result, actin filament breakdown is restrained and polymerization becomes dominant. Functionally, this shift is expected to increase mechanical robustness and resistance to deformation, whether the relevant stressor is synaptic activity in the hippocampus or elevated intraglomerular pressure in the kidney.

### 8.3. Cytoskeletal Protein Synthesis (PI3K/Akt/mTOR and MicroRNAs)

Via PI3K/Akt/mTOR, together with coupled CREB-dependent programs, BDNF/TrkB can enhance protein synthesis in neurons and support growth and remodeling of the dendritic arbor [46]. BDNF-linked miRNA regulation of the LIMK1–cofilin module (including miR-132/miR-134) is discussed in detail in Section 6 and is not repeated here.

In neurons, greater structural stability of the actin network within spines is required to anchor key receptors, such as AMPA receptors, that support memory consolidation and synapse stabilization [88]. Without a stable actin scaffold, synaptic signaling becomes less durable, and this state has been linked to depressive phenotypes and cognitive impairment [89,90].

In podocytes, the logic is analogous, but the functional outcome is preservation of the glomerular filtration barrier rather than memory consolidation [80]. Under elevated pressure, exposure to toxins, or inflammatory cues, podocytes are prone to foot process effacement and weakened attachment to the glomerular basement membrane. By promoting actin polymerization and suppressing cofilin activity, BDNF may help podocytes maintain stiffness and adhesion, which has been associated with reduced proteinuria in experimental models [23].

Taken together, BDNF can be viewed as a broadly conserved component of cellular responses to mechanical and metabolic stress. Across tissues, it appears to implement a shared strategy by stabilizing the actin scaffold and increasing cellular mechanical endurance.

If BDNF/TrkB signaling does enhance the mechanical robustness of these structures, the most informative test bed would be conditions in which the glomerulus is exposed to maximal hemodynamic and metabolic load. For this reason, the next section considers functional stress during extreme endurance exercise as a natural model of a peak challenge to the brain–kidney axis.

## 9. Systemic Effects of Extreme Exercise on the Brain–Kidney Axis

Despite the well-established benefits of regular, moderate-intensity aerobic activity reflected in WHO recommendations [91], certain exercise formats can impose pronounced acute functional stress [28]. Athletes in extreme endurance disciplines such as ultramarathon, triathlon, and trail running are exposed to a convergent stress load that combines thermal strain, hypovolemia due to dehydration, metabolic stress, inflammation, and activation of the HPA and the sympathetic nervous system. When events are performed at altitude, hypoxic stress adds an additional challenge.

Such exposure affects multiple target organs (e.g., the kidneys). Across several studies, a substantial proportion of ultra-endurance participants show transient increases in creatinine, NGAL, and urea that fall within the spectrum of exercise-associated renal stress. In a subset of athletes, these changes meet criteria for AKI based on creatinine kinetics and biomarker dynamics [28,92,93,94,95]. In parallel, circulating levels of BDNF, VEGF, and other mediators also change, likely reflecting components of a systemic adaptive response to neuroendocrine activation, inflammatory signaling, and altered vascular reactivity [96,97]. When interpreting BDNF dynamics, the assay matrix is critical, since platelet activation and the coagulation procedure can markedly shift absolute concentrations, with serum values typically exceeding plasma values [22,26].

Several prolonged exercise studies report an acute rise in circulating BDNF that is likely driven, at least in part, by release from the platelet reservoir during platelet activation. By contrast, the contribution of skeletal muscle to circulating BDNF remains debated and appears limited [98,99,100,101]. Conceptually, this transient increase could increase BDNF availability to peripheral tissues. For the glomerulus, the key question is how much of this signal reaches podocytes and which source dominates, local renal production or the systemic circulating pool. Under combined stress exposures that include heat, dehydration, NSAID use, endothelial activation, and impaired endothelium-dependent vasoreactivity, any protective contribution of BDNF and TrkB signaling may be attenuated and may coincide with greater vulnerability to exercise-associated renal stress [28,29].

Extreme endurance efforts can also be accompanied by transient signs consistent with blood–brain barrier (BBB) disruption and short-lived changes in cognitive performance in a subset of participants [102,103,104,105]. In several studies, exercise-induced increases in serum S100B have been interpreted as being compatible with altered BBB function in the context of an intensified systemic inflammatory milieu [106,107]. However, S100B is not brain-specific, and exercise-associated elevations may also reflect extracranial release, including contributions from skeletal muscle damage and adipose tissue–related mechanisms [108,109,110].

Given these limitations, complementary blood biomarkers with higher CNS relevance may be considered to contextualize CNS stress, such as glial fibrillary acidic protein (GFAP) as an astroglial injury/stress marker and neurofilament light chain (NfL) as an indicator of neuroaxonal injury, while recognizing that neither biomarker is specific for BBB permeability and both can be influenced by systemic confounders [111,112]. In parallel, circulating brain-derived extracellular vesicles and related particles, which retain membrane components and cargo reflecting their cells of origin, have been proposed as “liquid biopsy” candidates for CNS states and may offer an additional readout of neurovascular interface perturbation in disease or extreme physiological stress [113,114]. In this context, immunocapture/enrichment strategies using neuron-associated surface proteins (e.g., L1CAM) have been widely used, although methodological caveats and specificity considerations should be acknowledged [115,116].

Finally, BBB-disrupting stimuli have been associated with extracellular microvesicle shedding that can carry tight-junction proteins (e.g., claudin-5, occludin), providing a mechanistically plausible “membrane-fragment” signal in blood, although translation to exercise settings requires further validation [117,118]. The broader implication is that under extreme stress, endothelial and barrier systems may be particularly vulnerable, which is consistent with increased microvascular strain and heightened stress exposure of target organs, including the glomerulus.

Proposed mechanisms underlying increased BBB permeability under these conditions are multifactorial. They may include amplified inflammation and oxidative stress, with increased pro-inflammatory cytokines such as TNF-α, IL-1β, and IL-6 that can impair endothelial function [119]. Additional contributors include hyperthermia and dehydration, which increase plasma osmolality and may weaken tight-junction integrity [120,121], peripheral vasoactive and neuromediator influences including potential serotonergic effects on the endothelium [122], and episodes of reduced cerebral perfusion or hypoxemia during extreme exercise as part of the broader systemic stress response [123]. Among the proposed contributors, the most consistent evidence in the exercise literature relates to systemic inflammation/oxidative stress and thermal–hydration strain (hyperthermia and hypohydration), which can converge on endothelial tight-junction regulation [119,120,121]. Altered perfusion/oxygenation is also plausible during extreme exposures, particularly when combined with heat stress or altitude, but evidence is more context-dependent [123]. By contrast, receptor-specific neuromodulatory mechanisms (e.g., serotonergic effects on the endothelium) appear more speculative in the specific setting of endurance exercise and should be presented as lower-certainty contributors [122]. At the behavioral level, psychological fatigue and sickness behavior are frequently reported and are commonly attributed to systemic inflammatory activation. Elevated pro-inflammatory cytokines are thought to influence the brain through endothelial and neuroimmune mechanisms. Transient BBB permeability shifts have been reported in some extreme-exercise contexts. If present, they could in principle increase the likelihood that circulating mediators gain greater access to CNS-facing interfaces (e.g., endothelial signaling and perivascular routes) [106,107]. Whether this translates into measurable changes in behavior or cognitive performance likely depends on the magnitude and duration of permeability changes and the concurrent inflammatory and thermal milieu, and should therefore be interpreted as a hypothesis rather than an established causal chain [124].

From a brain–kidney axis perspective, extreme endurance exercise is a complex functional stressor that increases hemodynamic and metabolic load on the glomerulus, engages inflammatory and endothelial pathways, and alters the availability of systemic mediators, including BDNF. In most cases, these responses are short-lived and reversible. However, when exposures are repeated or recovery is insufficient, they may set the stage for recurrent renal stress and transient shifts in vascular, endothelial, and barrier regulation. If, during extreme exercise, BDNF is viewed as part of an adaptive stress response, clinical observations in chronic kidney disease raise a related question. How does chronic nephron injury reshape BDNF and TrkB signaling, and is any podocyte-level protective role preserved under these conditions?

## 10. BDNF in Kidney Disease

CKD represents a sustained injury milieu characterized by persistent inflammation, endothelial dysfunction, hypoxia-related signaling, metabolic disturbances, and structural remodeling. Therefore, parallels with acute exercise stress should be treated as hypothesis-generating rather than directly translatable across pathophysiological regimes. In this context, the BDNF/TrkB axis should be interpreted as a context-dependent stress-response pathway that may participate in adaptive repair processes but can also intersect with maladaptive remodeling depending on cell type, exposure intensity, and duration.

In CKD, BDNF/TrkB axis components have been reported in kidney-relevant cellular compartments, supporting the concept that nephron and vascular cells can engage local autocrine/paracrine signaling loops during injury responses [24]. Mechanistically, such local signaling could influence cytoskeletal stability, junctional organization, and repair programs, particularly in glomerular cells and the renal endothelium, where structural resilience is critical for maintaining filtration barrier function.

Physical activity is increasingly viewed as a modifier of BDNF biology. In rehabilitation studies of moderate aerobic exercise in CKD populations, including dialysis cohorts, increases in circulating BDNF have been discussed primarily in relation to neurocognitive outcomes and quality of life [125,126]. From a renal-mechanistic perspective, these observations motivate a cautious hypothesis that repeated moderate exercise may support resilience pathways, while acknowledging that blood BDNF is strongly influenced by platelet biology and systemic confounding and therefore does not directly report renal effector signaling [22,98,101]. Importantly, prospective human data linking exercise-associated BDNF dynamics to podocyte-centered injury markers and long-term renal outcomes remain limited [28].

Experimental glomerular injury models provide mechanistic support for a TrkB-dependent cytoskeletal resilience program, including PI3K/Akt signaling, mTOR engagement, and actin-regulatory nodes such as LIMK1–cofilin, with reported associations with preserved foot process architecture and reduced proteinuria [23,80]. However, translating these findings to human CKD requires caution because isoform-specific TrkB contributions, dominant BDNF sources (local vs. systemic), and operating conditions under chronic injury remain incompletely defined.

Despite important uncertainties in source attribution and causality, the available literature supports several points with reasonable confidence. First, components of the BDNF/TrkB axis are detectable in renal tissue and show dynamic regulation in chronic injury contexts, consistent with stress-responsive signaling programs. Second, experimental evidence in glomerular injury models supports a capacity of BDNF/TrkB signaling to engage actin-regulatory pathways relevant to podocyte structural integrity and proteinuria-related outcomes, although isoform-specific contributions remain unresolved. Third, in CKD the interpretation of circulating BDNF is strongly confounded by platelet biology, inflammation, and comorbid vascular disease. Therefore, mechanistic inference should prioritize kidney-compartment signals and cell-type context over systemic levels.

## 11. Translational Perspectives

From a translational perspective, the immediate value of the BDNF/TrkB framework is to support risk stratification, mechanism-informed monitoring, and testable intervention hypotheses in physically stressed individuals. A pragmatic starting point is to identify conditions in which exercise becomes a renal threat—heat stress and dehydration, repeated high-load events with insufficient recovery, NSAID exposure, rhabdomyolysis risk, and vascular vulnerability (e.g., increased arterial stiffness and elevated pulse pressure) and to treat these as modifiers of glomerular mechanical burden and podocyte stress [28,34,35].

Because circulating BDNF is strongly shaped by platelet biology and sampling methodology, translational studies should prioritize paired blood matrices (e.g., serum and platelet-poor plasma) alongside renal endpoints [22,25,26]. Pre-specifying matrix choice and platelet control is essential for interpretability [25,26,27]. The most informative designs are likely to combine functional measures (creatinine kinetics/eGFR, albuminuria/uACR) with injury and compartment-focused biomarkers (tubular stress markers and podocyte-centered readouts, including urinary sediment transcript profiling where feasible), collected across repeated stress exposures and recovery trajectories [28]. Such datasets can help distinguish whether BDNF-related changes track systemic stress/platelet activation, local renal responses, or both [22,25,26,27].

While BDNF-targeted therapy is not established for exercise-related renal stress, several actionable domains can be evaluated within standard sports medicine and nephrology frameworks: (a) hydration and heat-mitigation strategies to reduce hemoconcentration and thermal strain; (b) avoidance of NSAIDs around high-risk events; (c) training and competition scheduling that limits repeated exposures without adequate recovery; and (d) individualized risk counseling for athletes with known vascular or renal vulnerability [28]. These measures primarily reduce hemodynamic and inflammatory load on the filtration barrier and can be assessed with podocyte- and tubule-centered endpoints [28,34,35,36].

The strongest mechanistic rationale currently resides in experimental glomerular injury models indicating that BDNF/TrkB signaling can engage actin-regulatory pathways relevant to podocyte structural integrity and proteinuria outcomes [23,80]. Translationally, this suggests two testable directions, enhancing local renal resilience signaling (rather than relying on circulating BDNF) and clarifying isoform-specific TrkB actions in glomerular cell types to define targetability [23]. However, any strategy that chronically amplifies BDNF/TrkB signaling warrants caution, because sustained pathway activation could, in principle, shift cytoskeletal remodeling toward maladaptive states or reduce the ability of podocytes to adapt to dynamic mechanical demands [23,80]. Therefore, safety assessment should be integrated early, with attention to dose, duration, cell-type specificity, and context dependence.

Overall, the translational priority is to move from associative observations to causal, endpoint-driven designs that map BDNF source, bioavailable fraction, receptor isoforms, and glomerular targets under defined stress exposures [23,28]. This will clarify whether BDNF/TrkB functions primarily as a context-dependent biomarker of systemic stress, a local renal resilience module, or a modulator bridging central stress circuits and podocyte stability [22,23,25,26,27].

## 12. Limitations

A major limitation is unresolved source attribution as it remains unclear whether podocyte signaling is driven primarily by local renal BDNF versus the bioavailable circulating fraction and by which delivery routes. A second limitation is that podocyte expression data for truncated TrkB isoforms (e.g., TrkB-T1) are more robust than isoform-specific functional validation. Thus, receptor–isoform attribution of calcium and cytoskeletal effects in podocytes remains provisional. Circulating BDNF is strongly confounded by platelet-associated pools and sampling methodology (serum vs. plasma/platelet-poor plasma), limiting inference about the free fraction available for renal effector signaling. Finally, species- and model-dependence (including mRNA vs. protein readouts and injury-model context) limits direct extrapolation from experimental systems to human exercise-related renal stress.

## 13. Conclusions

Functional stress during extreme endurance exercise can transiently shift renal physiology toward a vulnerable state, particularly when compounded by dehydration/heat strain, vascular stiffness, NSAID exposure, and insufficient recovery. In this setting, the glomerular filtration barrier, and podocytes in particular, represents a plausible cellular bottleneck because podocyte function critically depends on actin cytoskeletal stability under hemodynamic and metabolic load. BDNF/TrkB signaling is a biologically plausible candidate pathway linking systemic stress adaptation to cytoskeletal resilience programs, given its established roles in actin-dependent structural plasticity and stress-related neuroendocrine regulation, and mechanistic support for protective actions in renal compartments in experimental injury contexts.

At the same time, the current evidence base remains insufficient to assign a dominant causal route for BDNF within the brain–kidney axis during exercise-related renal stress. Therefore, translational interpretation should prioritize source attribution (local vs. circulating), the bioavailable fraction reaching glomerular cells, and isoform-specific TrkB actions before BDNF/TrkB can be considered beyond a candidate modulatory axis in exercise-related renal stress. Key uncertainties include the relative contributions of local renal versus circulating BDNF, the effective availability of free BDNF to glomerular cells under physiological conditions, and isoform-specific TrkB roles (full-length versus truncated variants) in podocytes and neighboring glomerular cell types. Moreover, circulating BDNF measurements are strongly influenced by platelet-associated pools and methodological factors (e.g., serum vs. plasma), limiting direct inference about effector signaling in the kidney.

Future progress will require integrative, endpoint-driven designs that combine source attribution and receptor isoform profiling with podocyte-centered outcomes under defined stress exposures. In humans, the most informative approaches are likely to include paired blood matrices (serum and platelet-poor plasma), urinary sediment transcript profiling, and longitudinal assessment of glomerular injury markers alongside renal functional measures across repeated exercise stressors. Together, such studies could enable causal testing of whether BDNF/TrkB acts primarily as a context-dependent biomarker of systemic stress, a local renal resilience module, or a modulatory bridge between central stress circuits and glomerular barrier stability.

## Data Availability

No new data were created or analyzed in this study.

## Abbreviations

AKI	acute kidney injury
BBB	blood–brain barrier
BDNF	brain-derived neurotrophic factor
CKD	chronic kidney disease
CNS	central nervous system
CRH	corticotropin-releasing hormone
GFAP	glial fibrillary acidic protein
HPA	hypothalamic–pituitary axis
NfL	neurofilament light chain
NSAID	nonsteroidal anti-inflammatory drug
TrkB	tropomyosin receptor kinase B
TrkB-FL	tropomyosin receptor kinase B (full-length)
TrkB-T1, TrkB-T2	tropomyosin receptor kinase B (truncated)
uACR	urine albumin-to-creatinine ratio
